# *T. marneffei* infection complications in an HIV-negative patient with pre-existing pulmonary sarcoidosis: a rare case report

**DOI:** 10.1186/s12879-018-3290-7

**Published:** 2018-08-10

**Authors:** Xiaoming Yu, Keji Miao, Changsheng Zhou, Yuelin Cai, Xiaoying Huang, Yanfan Chen, Mayun Chen, Hui Cai, Lin Zhang

**Affiliations:** 1 Division of Pulmonary Medicine, the People’s Hospital of Cangnan, Wenzhou Medical University, No.2288, Yucang Road, Cangnan County, Zhejiang, 325800 China; 2 Division of Pulmonary Medicine, First Affiliated Hospital of Wenzhou Medical University, Key Laboratory of Heart and Lung, Wenzhou, Zhejiang, 325000 China; 3 Division of Pulmonary Medicine, Zhongshan Hospital, Fudan University, Shanghai, 200032 China

**Keywords:** *Talaromyces marneffei*, HIV-negative, Pulmonary sarcoidosis

## Abstract

**Background:**

*Talaromyces marneffei* (*T. marneffei*) is a thermal dimorphic pathogenic fungus that often causes fatal opportunistic infections in human immunodeficiency virus (HIV)-infected patients. Although *T. marneffei-*infected cases have been increasingly reported among non-HIV-infected patients in recent years, no cases of *T. marneffei* infection have been reported in pulmonary sarcoidosis patients. In this case, we describe a *T. marneffei* infection in an HIV-negative patient diagnosed with pulmonary sarcoidosis.

**Case presentation:**

A 41-year-old Chinese man who had pre-existing pulmonary sarcoidosis presented with daily hyperpyrexia and cough. Following a fungal culture from bronchoalveolar lavage (BAL), the patient was diagnosed with *T. marneffei* infection. A high-resolution computed tomography (HRCT) chest scan revealed bilateral lung diffuse miliary nodules, multiple patchy exudative shadows in the bilateral superior lobes and right inferior lobes, air bronchogram in the consolidation of the right superior lobe, multiple hilar and mediastinal lymphadenopathies and local pleural thickening. After 3 mos of antifungal therapy, the patient’s pulmonary symptoms rapidly disappeared, and the physical condition improved markedly. A subsequent CT re-examination demonstrated that foci were absorbed remarkably after treatment. The patient is receiving follow-up therapy and assessment for a cure.

**Conclusion:**

This case suggested that clinicians should pay more attention to non-HIV-related lung infections in patients with pulmonary sarcoidosis. Early diagnosis and treatment with antifungal therapy can improve the prognosis of *T. marneffei* infection.

## Background

*Talaromyces marneffei (Penicillium marneffei*), first discovered in 1956, is a thermally dimorphic fungus that can cause severe infections in epidemic regions of Southeast Asia, particularly in immunocompromised patients [1–3]. It develops into a mycelium at 25 °C and into yeast at 37 °C, but only the yeast-like form has pathogenic potential. Patients with HIV/AIDS have been reported to be vulnerable to *T. marneffei* [4, 5]. However, a growing number of *T. marneffei*-infected patients without HIV have been reported in recent years [6–8]. Among non-HIV-infected patients, to our knowledge, those who suffer from long-standing pulmonary sarcoidosis have rarely been reported to be subject to *T. marneffei* infection. We herein describe the details of the first such case worldwide.

## Case presentation

**Fig. 1:**
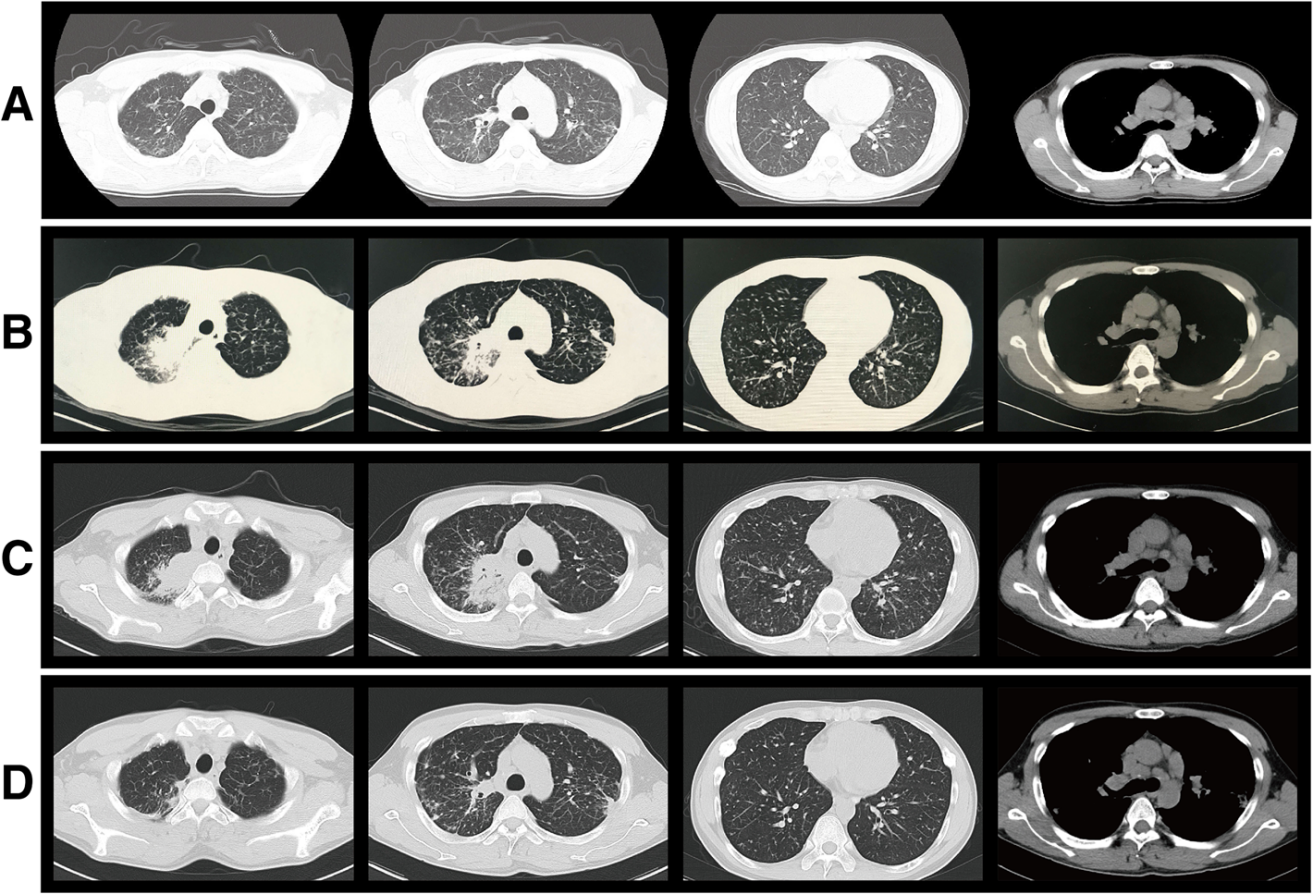
**a** Past chest CT scan (February 20, 2015) showing bilateral lung diffuse miliary nodules. **b** Chest CT scan on day 10 before hospital admission (March 31, 2017) showing plaques and nodules disseminated throughout the bilateral lung. **c** Chest CT scan on day 2 of hospital admission (April 12, 2017) showing bilateral lung diffuse miliary nodules, multiple patchy exudative shadows in the bilateral superior lobes and right inferior lobes, and an air bronchogram in the consolidation of the right superior lobe. **d** Chest CT scan during follow-up (July 18, 2017) showing the absorption of lesions and nodules

**Fig. 2:**
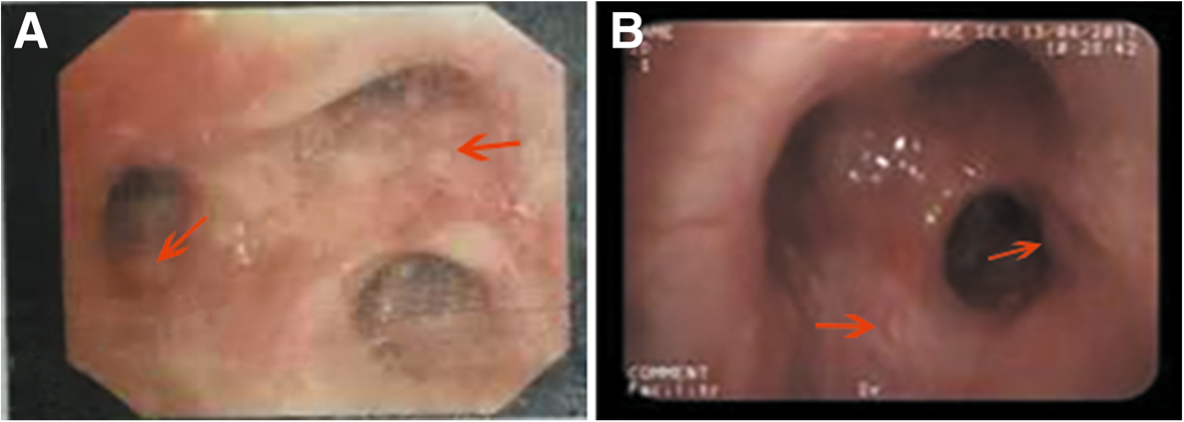
Bronchoscopic examinations revealed a nodular projection on the surface of the right apical bronchus. **a** Examination on March 31, 2017 in a hospital in Shanghai. **b** Examination on April 13, 2017 in the People’s Hospital of Cangnan (Red arrows: nodules)

**Fig. 3:**
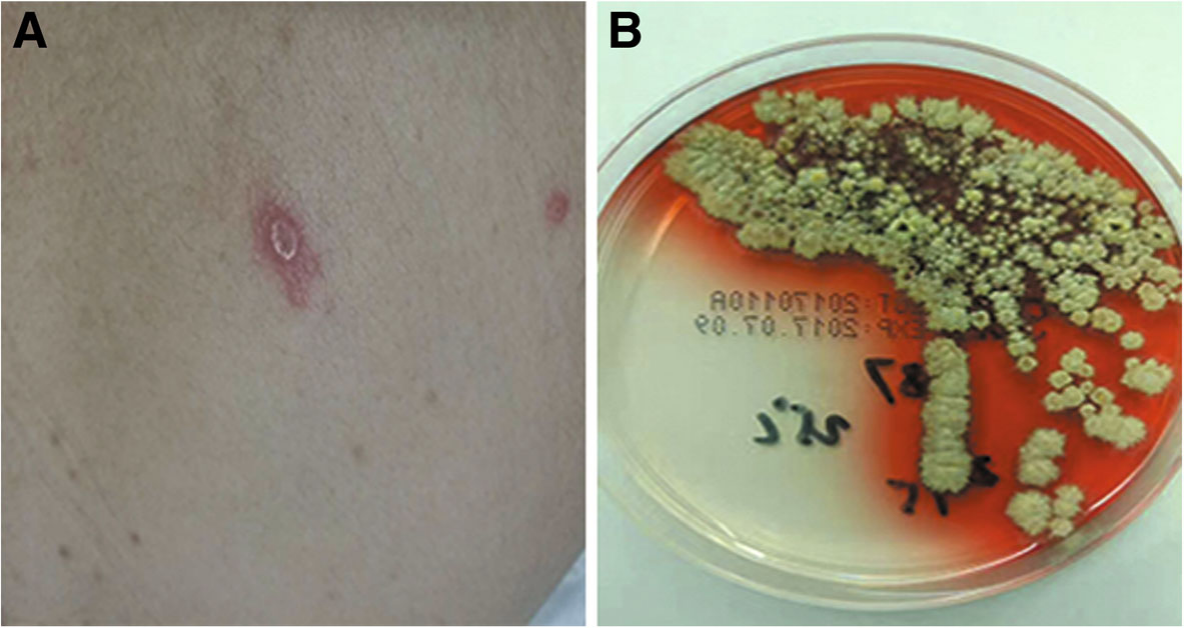
**a** The skin lesions on the patient’s back. **b** Culture of *Talaromyces marneffei* from our patient with distinctive red diffusible pigment (after 7 days incubation at 25 °C, Sabouraud agar)

A 41-year-old man, a native of Cangnan County in the Zhejiang province of southeast China, was admitted to our hospital because of a 3-week history of daily hyperpyrexia and sputum-coughing in April 2017. The first time that multiple pulmonary nodules and bilateral hilar lymphadenopathy were found in chest CT (Fig. 1a) was 7 years ago. The patient was diagnosed with pulmonary sarcoidosis according to the results of a transbronchial needle aspiration (TBNA) and transbronchial lung biopsy (TBLB), which revealed lymphocytes, columnar epithelial cells and a cloud of epithelial-like cells. In the following years, he received follow-up chest CT examination and corticosteroid treatment irregularly. The patient met the ATS/WASOG diagnostic criteria for sarcoidosis because there was no progression of the lesions in recent years. With the pre-existing pulmonary sarcoidosis, he had been diagnosed with the progression of pulmonary sarcoidosis in a certain hospital in Shanghai 12 days prior. At that time, he was examined with chest CT and central ultrasound bronchoscopy. The chest CT showed space-occupying lesions of the right superior lobe, probably a malignant tumour, mediastinal and right hilum lymphadenopathy, and plaques and nodules disseminated throughout the bilateral lung, probably pneumoconiosis and metastasis (MT) (Fig. 1b). Compared to the initial chest CT performed in 2015 (Fig. 1a), Fig. 1b shows increased miliary pulmonary nodules and a new pulmonary consolidation. Central ultrasound bronchoscopy revealed that a nodular projection was on the surface of both superior lobar bronchus and that stenosis appeared in the right superior lobar bronchus, especially the right apical segment (Fig. 2a). The patient received transbronchial needle aspiration (TBNA) 6 times when the ultrasound probed a tumour outside of the right primary bronchus and lymphadenectasis in 11R and 10 L. The pathology exam found fibrous tissue hyperplasia accompanied by apparent infiltration of monocytes and lymphocytes. There was no evidence of non-caseating epithelioid granuloma. Moreover, eosinophils were infiltrated in some areas. After 3 days of prednisone and levofloxacin, the fever and cough persisted, and there was no clinical improvement; even worse, skin lesions (Fig. 3a) erupted on his back.

The patient was pale and had intermittent high fever on the day of admission to our hospital. Born and raised in Cangnan, he denied a residential history in any epidemic regions. After his admission, imipenem cilastatin was promptly used against the infection. Immediate HRCT revealed bilateral lung diffuse miliary nodules, multiple patchy exudative shadows in the bilateral superior lobes and right inferior lobes, air bronchogram in the consolidation of the right superior lobe, multiple hilar and mediastinal lymphadenopathies and local pleural thickening (Fig. 1c). The image did not change much compared to the previous one. The results of the following laboratory routine examinations are shown in Table 1. Microbiology analysis showed that repeated sputum smears, sputum culture, and blood cultures were negative. Combining these clinical manifestations and biochemical analyses, the chance of tuberculosis diagnosis could not be excluded. However, the T-spot was negative, and acid-fast bacilli were not present. It was difficult to explain the extremely elevated IgE and eosinophils in the blood as well as the eosinophil infiltration in the bronchus on the monism of tuberculosis. After providing informed consent, the patient underwent bronchoscopy again, which revealed an unevenness of the trachea in the subglottic region as well as protrusions on the tracheal wall, especially in the right superior lobar bronchus (Fig. 2b). A subsequent (1/3) -b-D-glucan (G) assay was positive, and a smear was positive for fungus in the BAL. All the evidence above was associated with a higher likelihood of fungal infection. Along with the evidence that there was no clinical improvement after 4 days of antibacterial therapy, the patient was suspected of pulmonary aspergillosis because it was endemic in the region of Cangnan. He was immediately treated with voriconazole at a dosage of 200 mg/dL every 12 h via intravenous administration starting on April 14, 2017. Ultimately, the fungal culture of bronchoalveolar confirmed the diagnosis of *T. marneffei* infection on April 20 (Fig. 3b). His fever returned to normal, and his respiratory signs disappeared gradually after an 8-day treatment, as well as his skin lesions. The results of the chest CT re-examination showed that the lung lesions were markedly absorbed after 3 months (Fig. 1d). He continued to receive follow-up antifungal treatment.

**Table 1:** Laboratory Findings at First Presentation

glucose	4.34 g/L	AFP	4.2 ng/ml
T-BIL	14.0 μmol/L	CA724	0.9 U/ml
GGT	420 g/L	CYRA21–1	2.1 ng/ml
ALT	92 μmol/L	WBC	8.46 × 10^9^ /L
AST	60 μmol/L	Neu	68.3%
BUN	4.51 g/L	Lym	8.0%
SCr	59 g/L	EOS	17.4%
CRP	114.84 mg/L	Hb	124 g/L
PCT	1.840 ng/ml	MCV	84.6 fl
HIV-Ab	(−)	PLT	300 × 10^9^ /L
SCC	0.4 ng/mL	Serum G assay	(−)
NSE	22.1 ng/ml	Serum GM assay	(−)
Pro-GRP	28.3 pg/mL	T-spot	(−)
CEA	0.9 U/ml	Total IgE	>2500.00 KIU/L
CA125	41.6 U/ml	BAL-G assay	(+)
CA199	12.5 U/ml	BAL-GM assay	(−)

*T-BIL* Total- bilirubin, *GGT* gamma-glutamyl transpeptidase, *ALT* alanine transaminase, *AST* aspartate transaminase, *BUN* blood urea nitrogen, *SCr* serum creatinine, *CRP*=C-reactive protein, *PCT* procalcitonin, *SCC* squamous cell carcinoma antigen, *NSE* neuron specific endase, *pro-GRP* Human pro-gastrin-reliasing peptide, *CEA* carcinoembryonic antigen, *CA125* carbohydrate antigen 125, *CA199* carbohydrate antigen 199, *AFP* alpha fetoprotein, *CA724* carbohydrate antigen 724, *CYRA21–1* cytokeratin 19 fragment, *WBC* white blood cell count, *Neu* neutrophil count, *Lym* lymphocyte count, *EOS* eosnophils, *Hb* hemoglobin, *MCV* Mean Corpuscular Volume, *PLT* platelets, *G assay* (1/3) -b-D-glucan assay; *GM assay* galactomannan antigen assay

## Discussion

*Talaromyces marneffei*, the only known dimorphic fungus of the genus Penicillium, was first isolated in 1956 in Vietnam from the bamboo rat *Rhizomys sinensis* [9]. A diagnostic characteristic of *T. marneffei* is mould-to-yeast conversion or phase transition, which is thermally regulated. Since the first natural *T. marneffei* infection was reported in 1973 [10], it has been increasingly observed both in AIDS patients and in HIV-negative individuals in recent years. Among non-HIV-infected individuals, pulmonary *T. marneffei* infection has been reported in patients with a history of pulmonary tuberculosis [6] or chronic obstructive pulmonary disease (COPD) [11]. However, to our knowledge, the infection has not been reported in patients with a history of pulmonary sarcoidosis. In this article, we first present such a case of a confirmed diagnosis of *T. marneffei* infection in a non-HIV-infected patient with pre-existing pulmonary sarcoidosis.

The main route of transmission of *T. marneffei* is inhaling the infectious agent; rarely is there direct animal contact. The typical clinical manifestations are fever, weight loss, skin lesions, generalized lymphadenopathy, hepatosplenomegaly, and respiratory signs, but the severity of the disease depends on the patient’s immune status [12, 13]. The patient in this case was a non-HIV-infected patient and was young, but his lung immunity was probably impaired due to long-standing pulmonary sarcoidosis. Early in 1988, Deng, Z. et al. [14] reported that southern China was one of the endemic regions for *T. marneffei*. Specifically, these clinical features, as indicated by hyperpyrexia, sputum-coughing, persistent elevated IgE and eosinophils in the blood, eosinophil infiltration in the bronchus, positive BAL-G assay (G assay of BAL), and T-spot negativity as well as the failure to reveal acid-fast bacilli, led to possible infection with a pulmonary fungus. As the accuracy of the BAL-G assay is marginal rather than absolutely specific for invasive fungal disease (IFDs), the results should not be interpreted alone but should be used as a part of a full assessment together with clinical features, image findings and other laboratory results for the diagnosis of IFDs [15]. Finally, the *T. marneffei* infection was confirmed with bronchoalveolar lavage culture. In addition to the BAL, commonly used clinical specimens in the literature include bone marrow aspirate, blood, lymph node biopsies, skin biopsies, skin scrapings, sputum, pleural fluid, liver biopsies, cerebrospinal fluid, pharyngeal ulcer scrapings, palatal papule scrapings, urine, stool samples, and kidney, pericardium, stomach or intestine specimens [16]. Different from previously reported cases of pulmonary *T. marneffei* infection in non-HIV-infected patients, the pre-existing pulmonary sarcoidosis covered up the clinical futures of *T. marneffei* and easily misled us about the progression of the original disease or lymphoma.

The *T. marneffei* presentation upon chest CT is non-specific, as displayed by multiple patchy exudative shadows, pulmonary consolidation, nodular shadows, a ground-glass appearance, miliary lesions, and nodular masses, commonly accompanied by mediastinal and hilum lymphadenopathy and sometimes by cavitary lesions [17]. Compared to the chest CT (Fig. 1a) 2 yrs prior, the chest CT (Fig. 1b and c) showed the progression of pulmonary nodules and the new consolidation lesions. In this respect, we would be more likely to suspect the progression of pulmonary sarcoidosis and to ignore the possibility of fungal infection. The chest CT (Fig. 1d) re-examined after anti-fungal treatment for 3 months showed that the lung lesions as well as some pulmonary nodules were markedly absorbed. However, the mediastinal lymphadenopathy did not improve in all the groups, as shown in Fig. 1. There was a strong likelihood that the lymphadenopathy was due to long-standing pulmonary sarcoidosis.

The non-specific presentation of *T. marneffei* highlights the importance of the rapid diagnosis and treatment of this potentially life-threatening mycosis. *T. marneffei* is susceptible to itraconazole and amphotericin B in vitro [18, 19]. A study from China revealed that voriconazole had the lowest MIC (ranged from 0.004 mg/L to 0.25 mg/L) in comparison to other antifungal agents, and the results showed that voriconazole and itraconazole are active against *T. marneffei* isolated in vitro [20]. However, a documented study reported that a single dose of itraconazole for the treatment of *T. marneffei* infection in HIV-infected patients was non-effective [21]. A retrospective study evaluating the efficacy and safety of voriconazole to treat patients with *T. marneffei* infection suggested that voriconazole was an effective, well-tolerated therapeutic option for this disease [22]. Taken together, we preferred voriconazole as the antifungal drug for this case. Indeed, the patient recovered rapidly, and the lung lesions were markedly absorbed after treatment.

## Conclusions

In summary, our study reports a case of *T. marneffei* infection in a non-HIV-infected patient with a history of pulmonary sarcoidosis in an endemic fungal area. This study invites clinicians to consider *T. marneffei* infection in non-HIV-infected patients with underlying diseases because early diagnosis and proper treatment lead to a reduction in the mortality associated with *T. marneffei*.

## Abbreviations

BAL: Bronchoalveolar lavage
G assay: (1/3) -b-D-glucan assay
GM assay: Galactomannan antigen assay
HIV: Human immunodeficiency virus
HRCT: High-resolution computed tomography
*T. marneffei*: *Talaromyces marneffei*
TBNA: Transbranchial needle aspiration
